# Virulence determinants in multidrug-resistant *Acinetobacter baumannii*: comparative phenotypic and genomic analysis of clinical and patient-environmental isolates

**DOI:** 10.3389/fcimb.2026.1969523

**Published:** 2026-10-16

**Authors:** Preethi Basavaraju, Rashmi P. Mahale, Morubagal Raghavendra Rao, Suchitra Shenoy M

**Affiliations:** 1 Department of Microbiology, JSS Medical College & Hospital, JSS Academy of Higher Education & Research, Mysuru, Karnataka, India; 2 Department of Microbiology, Kasturba Medical College Manglore, Manipal Academy of Higher Education, Manipal, India

**Keywords:** *Acinetobacter baumannii*, biofilm, healthcare associated infection (HAI), hospital enviroment, multidrug resistance, virulence factor (VF), whole genome sequencing (WGS)

## Abstract

**Introduction:**

*Acinetobacter* baumannii is a major cause of healthcare-associated infections owing to its ability to acquire multidrug resistance and express multiple virulence determinants that facilitate persistence in both clinical and hospital environments. However, comparative studies evaluating the phenotypic expression of virulence factors alongside their genomic determinants in paired clinical and patient-environmental isolates remain limited. This study investigated the phenotypic virulence characteristics and corresponding genomic distribution of virulence-associated genes among multidrug-resistant (MDR) *A. baumannii* isolated from clinical specimens and patient environmental surroundings.

**Methods:**

A total of 110 non-duplicate MDR *A. baumannii* isolates, comprising 55 clinical and 55 corresponding patient-environmental isolates, were included. Biofilm formation, haemolytic activity, proteolytic activity, and siderophore production were evaluated using standard phenotypic assays. Whole-genome sequencing was performed on all isolates using the Illumina MiSeq platform, followed by genome assembly, annotation, and identification of virulence-associated genes using the Virulence Factor Database (VFDB). Comparative analyses were performed between clinical and environmental isolates.

**Results:**

Clinical isolates demonstrated significantly higher frequencies of strong biofilm formation (34/55, 61.8% vs. 6/55, 10.9%), haemolytic activity (29/55, 52.7% vs. 13/55, 23.6%), and siderophore production (31/55, 56.4% vs. 17/55, 30.9%) than environmental isolates (P < 0.05), whereas proteolytic activity was comparable between the two groups (36/55, 65.5% vs. 33/55, 60.0%). Whole-genome sequencing revealed a high prevalence of virulence-associated genes involved in biofilm formation, haemolysis, proteolysis, and iron acquisition among phenotypically positive isolates. Several virulence-associated genes were also detected in a subset of phenotypically negative isolates, indicating a phenotype–genotype discordance and suggesting that gene carriage alone does not necessarily predict phenotypic expression.

**Discussion:**

Clinical MDR *A. baumannii* isolates exhibited greater virulence than corresponding patient-environmental isolates; however, environmental isolates retained multiple virulence-associated genes, highlighting their potential role as reservoirs for persistence and transmission within healthcare settings. Integrating phenotypic virulence assays with whole-genome sequencing provides a comprehensive understanding of the virulence potential of MDR *A. baumannii* and underscores the importance of continuous environmental surveillance and infection prevention strategies to limit the dissemination of highly virulent strains.

## 1 Introduction

*Acinetobacter baumannii* has emerged as one of the most important opportunistic bacterial pathogens responsible for healthcare-associated infections worldwide. The organism predominantly affects critically ill and immunocompromised patients, causing ventilator-associated pneumonia, bloodstream infections, urinary tract infections, wound infections, and sepsis, particularly in intensive care units (ICUs). It’s remarkable ability to survive for prolonged periods on abiotic surfaces, acquire antimicrobial resistance determinants, and persist under adverse environmental conditions has made *A. baumannii* a major cause of hospital outbreaks and a significant challenge for infection prevention and control (Weber et al., 2010; Wong et al., 2017; Harding et al., 2018; Monem et al., 2020). Consequently, the World Health Organization (WHO) has designated carbapenem-resistant *A. baumannii* as a critical priority pathogen requiring urgent development of new therapeutic strategies and enhanced surveillance (Wong et al., 2017; Harding et al., 2018; World Health Organization, 2024).

The global increase in multidrug-resistant (MDR) *A. baumannii* has considerably limited available treatment options. Resistance to carbapenems is primarily mediated by the acquisition of carbapenem-hydrolysing class D β-lactamases (CHDLs), particularly the *bla*OXA-23 family, in addition to metallo-β-lactamases such as *bla*NDM (Wong et al., 2017; Harding et al., 2018; Sharma and Lakhanpal, 2025). Furthermore, the emergence of colistin resistance, one of the last-line therapeutic options for MDR *A. baumannii*, poses an additional threat to clinical management (Wong et al., 2017; Harding et al., 2018). However, antimicrobial resistance alone does not fully explain the remarkable success of *A. baumannii* as a nosocomial pathogen. The interaction between resistance mechanisms and bacterial virulence plays a crucial role in colonisation, persistence, immune evasion, and disease progression (Wong et al., 2017; Harding et al., 2018).

Virulence in *A. baumannii* is multifactorial and involves a diverse repertoire of factors that facilitate adaptation within both the host and the hospital environment (Wong et al., 2017; Harding et al., 2018). Biofilm formation is considered one of the important virulence traits, enabling bacterial adherence to biotic and abiotic surfaces, protection from host immune responses, and increased tolerance to antimicrobial agents (Espinal et al., 2012; Harding et al., 2018). Biofilm development is mediated by several genetic determinants, including the *csu* pili assembly operon, *ompA*, *bap*, the *pgaABCD* operon responsible for poly-β-1,6-N-acetylglucosamine synthesis, and quorum-sensing genes such as *abaI* and *abaR* (Choi et al., 2009; Harding et al., 2018; Oh and Han, 2020; Mea et al., 2021). These determinants contribute to bacterial persistence on medical devices and environmental surfaces, thereby facilitating prolonged survival within healthcare settings (Espinal et al., 2012; Harding et al., 2018).

In addition to biofilm formation, *A. baumannii* produces several extracellular virulence factors that enhance pathogenicity (Wong et al., 2017; Harding et al., 2018). Haemolytic activity contributes to host tissue damage and nutrient acquisition through haemolysins and phospholipase-associated mechanisms (Wong et al., 2017; Harding et al., 2018). Proteolytic enzymes, particularly CpaA, have been associated with degradation of host proteins and interference with host defence mechanisms (Tilley et al., 2014). Iron acquisition represents another essential virulence mechanism because iron availability within the host is tightly restricted. To overcome nutritional immunity, *A. baumannii* synthesises siderophores such as acinetobactin through coordinated activity of the *bas*, *bau*, and associated iron-acquisition genes, enabling efficient iron uptake and bacterial survival under iron-limited conditions (Dorsey et al., 2004; Sheldon and Skaar, 2020).

Whole-genome sequencing (WGS) has transformed the molecular epidemiology of *A. baumannii* by enabling simultaneous characterisation of antimicrobial resistance genes, virulence determinants, sequence types, and phylogenetic relationships with high resolution (Rafei et al., 2019) Compared with conventional molecular approaches targeting a limited number of genes, WGS provides a broader assessment of the genomic characteristics associated with bacterial adaptation and transmission (Wong et al., 2017; Harding et al., 2018). Integration of phenotypic virulence assays with genomic analysis further enables assessment of genotype–phenotype relationships and provides a more comprehensive understanding of the biological significance of virulence-associated determinants.

Despite increasing reports describing antimicrobial resistance in *A. baumannii*, comparatively fewer studies have comprehensively evaluated both phenotypic virulence characteristics and their corresponding genomic determinants among paired clinical and patient-environmental isolates. Hospital environmental reservoirs are recognised as important contributors to the persistence and potential transmission of MDR *A. baumannii*, particularly because of the organism’s ability to survive on dry and abiotic surfaces (Weber et al., 2010; Espinal et al., 2012; Monem et al., 2020). However, the extent to which environmental isolates retain virulence potential comparable to clinical isolates remains insufficiently understood, particularly in resource-limited healthcare settings.

Therefore, the present study aimed to investigate the phenotypic expression and genomic distribution of major virulence determinants among MDR *A. baumannii* isolated from clinical specimens and corresponding patient environmental surroundings in a tertiary care hospital in South India. Using a combination of conventional phenotypic assays and whole-genome sequencing, we characterised biofilm formation, haemolytic activity, proteolytic activity, and siderophore production together with their associated virulence genes, and compared their distribution between clinical and environmental isolates. This integrated approach provides insights into the relationship between virulence phenotype and genotype and enhances understanding of the persistence, pathogenic potential, and possible transmission dynamics of MDR *A. baumannii* within the hospital environment.

## 2 Materials and methods

### 2.1 Study design and ethical approval

A prospective hospital-based study was conducted in the Department of Microbiology, JSS Medical College and Hospital, JSS Academy of Higher Education & Research (JSSAHER), Mysuru, Karnataka, India, between August 2023 and October 2024. The study aimed to investigate the phenotypic virulence characteristics and genomic distribution of virulence-associated determinants among multidrug-resistant (MDR) *Acinetobacter baumannii* isolated from clinical specimens and corresponding patient environmental surroundings.

A total of 110 non-duplicate MDR *A. baumannii* isolates were included in the study, comprising 55 clinical isolates and 55 environmental isolates recovered from the surroundings of patients infected with MDR *A. baumannii*. Duplicate clinical isolates from the same patient and repeated environmental isolates with identical antimicrobial susceptibility profiles obtained from multiple sampling sites of the same patient environment were excluded.

The study was approved by the Institutional Ethics Committee of JSS Academy of Higher Education & Research (JSSAHER), Mysuru, India (Approval No. JSS/MC/PG/0040/2022–23). All procedures were performed in accordance with the Declaration of Helsinki and relevant institutional guidelines. Written informed consent was obtained from all participants before sample collection.

### 2.2 Collection of clinical and environmental isolates

Clinical specimens, including blood, urine, sputum, endotracheal aspirates, pus, wound swabs, body fluids, and other clinically indicated specimens, were processed according to standard microbiological procedures. Blood cultures were processed using the BacT/ALERT automated blood culture system (bioMérieux, Marcy-l’Étoile, France). Positive blood cultures and other clinical specimens were cultured on MacConkey agar and 5% sheep blood agar (HiMedia Laboratories, Mumbai, India), while urine specimens were cultured on Uri-Chrome agar (HiMedia Laboratories). Culture plates were incubated aerobically at 37 °C for 18–24 h. Semi-quantitative culture was performed for urine and endotracheal aspirate specimens.

Environmental sampling was performed for each patient from whom MDR *A. baumannii* had been isolated from a clinical specimen. Five environmental samples were collected from frequently touched surfaces surrounding the patient, including bed rails, bedside trolley, adjacent wall, floor surrounding the bed, and nursing station surfaces (Weber et al., 2010; Monem et al., 2020). Sterile cotton swabs pre-moistened with sterile normal saline were used for sample collection and transported immediately to the microbiology laboratory for processing.

A total of 275 environmental samples were collected from the surroundings of 55 patients. Based on identical antimicrobial susceptibility profiles, repeated environmental isolates obtained from multiple sites surrounding the same patient were excluded, resulting in a final collection of 55 non-duplicate environmental MDR *A. baumannii* isolates.

### 2.3 Isolation and identification of *Acinetobacter baumannii*

Clinical and environmental specimens were cultured on MacConkey agar and 5% sheep blood agar and incubated aerobically at 37 °C for 18–24 h. Presumptive *Acinetobacter* isolates were identified based on colony morphology, Gram staining, and conventional biochemical characteristics, including catalase positivity, oxidase negativity, and non-motility.

Clinical isolates were identified using the VITEK^®^ 2 Compact automated identification system with GN identification cards (BioMérieux, Marcy-l’Étoile, France). Environmental isolates were identified using conventional biochemical tests, including indole production, urease activity, citrate utilisation, and triple sugar iron (TSI) agar reactions. Species identification of all isolates was subsequently confirmed by whole-genome sequencing (Rafei et al., 2019).

### 2.4 Antimicrobial susceptibility testing

Antimicrobial susceptibility testing of clinical isolates was performed using the VITEK^®^ 2 Compact automated system with AST-N406 cards (BioMérieux, Marcy-l’Étoile, France). Environmental isolates were tested by the Kirby–Bauer disk diffusion method on Mueller–Hinton agar (HiMedia Laboratories) following the Clinical and Laboratory Standards Institute (CLSI) 2024 guidelines (Clinical and Laboratory Standards Institute, 2024). The antimicrobial panel included β-lactams, β-lactam/β-lactamase inhibitor combinations, carbapenems, aminoglycosides, fluoroquinolones, tetracyclines, folate pathway inhibitors, and other routinely tested antimicrobial agents. Isolates resistant to at least one antimicrobial agent in three or more antimicrobial classes were classified as multidrug-resistant (MDR) according to the international consensus criteria (Magiorakos et al., 2012). Only MDR *A. baumannii* isolates were included in the present study.

### 2.5 Phenotypic detection of virulence factors

The virulence potential of all MDR *Acinetobacter baumannii* isolates was assessed by evaluating four major phenotypic virulence characteristics, namely biofilm formation, haemolytic activity, proteolytic activity, and siderophore production. All assays were performed using freshly grown bacterial cultures under standard laboratory conditions.

#### 2.5.1 Biofilm formation

Biofilm formation was evaluated using the tissue culture plate (TCP) assay as described by Christensen et al. with minor modifications (Christensen et al., 1985). Briefly, isolates were inoculated into 5 mL of tryptic soy broth (TSB) and incubated at 37 °C for 24 h. The cultures were diluted 1:100 in fresh TSB, and 200 μL of the diluted suspension was dispensed into sterile 96-well flat-bottom polystyrene microtiter plates. Uninoculated broth served as the negative control. Following incubation at 37 °C for 24 h, the wells were gently washed twice with phosphate-buffered saline (PBS; pH 7.2), air-dried, and stained with 0.1% crystal violet for 10 min. Excess stain was removed by washing with distilled water, and the plates were allowed to dry. The bound crystal violet was quantified by measuring the optical density at 570 nm using a microplate reader (Bio-Rad Laboratories, Hercules, CA, USA). Biofilm production was classified according to the criteria described by *Stepanović* et al (Stepanović et al., 2007). as strong, moderate, weak, or non-biofilm producing. Weak biofilm producers were considered non-biofilm producers for subsequent analysis.

#### 2.5.2 Haemolytic activity

Haemolytic activity was determined by inoculating isolates onto 5% sheep blood agar (HiMedia Laboratories, Mumbai, India) followed by incubation at 37 °C for 24 h. Plates were examined for the presence of clear zones surrounding bacterial colonies. Isolates exhibiting complete haemolysis (β-haemolysis) were considered haemolysis-positive, whereas isolates showing no haemolysis (γ-haemolysis) were considered haemolysis-negative (Siau et al., 1998).

#### 2.5.3 Proteolytic activity

Proteolytic activity was evaluated using skim milk agar (HiMedia Laboratories, Mumbai, India). Fresh bacterial cultures were spot inoculated onto the agar surface and incubated at 37 °C for 24–48 h. Protease production was determined by the presence of a clear halo surrounding bacterial growth due to casein hydrolysis. Isolates without a visible zone of clearance were considered proteolysis-negative (Jones et al., 2007).

#### 2.5.4 Siderophore production

Siderophore production was assessed using Chrome Azurol S (CAS) agar (HiMedia Laboratories, Mumbai, India). Fresh bacterial isolates were inoculated onto CAS agar plates and incubated at 37 °C for 24–48 h. Siderophore production was identified by the appearance of an orange or yellow halo surrounding the colonies resulting from removal of ferric iron from the CAS dye complex. Isolates without colour change were considered siderophore-negative (Schwyn and Neilands, 1987).

### 2.6 Whole-genome sequencing and genomic analysis

Genomic DNA was extracted from all MDR *A. baumannii* isolates using the QIAamp DNA Mini Kit (Qiagen, Hilden, Germany) according to the manufacturer’s instructions. DNA concentration was determined using a Qubit Fluorometer (Thermo Fisher Scientific, Waltham, MA, USA) with the Qubit dsDNA BR Assay Kit. Sequencing libraries with an average insert size of approximately 450 bp were prepared using the Ultra FS II DNA Library Preparation Kit (New England Biolabs, Ipswich, MA, USA). Library quality and fragment size distribution were assessed using an Agilent TapeStation system (Agilent Technologies, Santa Clara, CA, USA), followed by paired-end sequencing on the Illumina MiSeq platform (Illumina, San Diego, CA, USA).

Raw sequencing reads were evaluated using FastQC for quality assessment (Andrews, 2010) and assembled *de novo* using SPAdes version 3.14 (Bankevich et al., 2012). Genome assemblies were annotated using Prokka version 1.5 (Seemann, 2014). Species identification was confirmed using BactInspector, and contamination was assessed using ConFindr (Low et al., 2019). Quality metrics were summarised using MultiQC (Ewels et al., 2016), and only high-quality genome assemblies were included for downstream analyses.

Core genome analysis was performed using Panaroo version 1.5.2 (Tonkin-Hill et al., 2020). Core genome single nucleotide polymorphisms (SNPs) were extracted using SNP-sites version 2.5.1 (Page et al., 2016), and a maximum-likelihood phylogenetic tree was constructed using IQ-TREE version 2.4.0 with 100 bootstrap replicates (Minh et al., 2020). Phylogenetic relationships were visualised using Microreact (Argimón et al., 2016). Virulence-associated genes present within the assembled genomes were identified using ABRicate version 1.0.0 against the Virulence Factor Database (VFDB) (Chen et al., 2005; Seemann, 2016), and the genomic findings were compared with the corresponding phenotypic virulence characteristics.

### 2.7 Statistical analysis

Statistical analyses were performed using GraphPad Prism version 10.0 (GraphPad Software, San Diego, CA, USA). Categorical variables were expressed as frequencies and percentages. Comparisons between clinical and environmental isolates were performed using the Chi-square test or Fisher’s exact test, as appropriate. A two-tailed *P* value of <0.05 was considered statistically significant.

## 3 Results

Our study entitled “Molecular characterization and genetic relatedness among multidrug resistant *Acinetobacter baumannii* from a tertiary care hospital” was carried out in the department of microbiology, JSS Medical college, to evaluate the molecular epidemiology and genetic relatedness among clinical and environmental isolates of Multi Drug Resistant (MDR) *Acinetobacter* spp.

The present study included, a total of 110 isolates of *Acinetobacter* spp. isolated during the study period of 15 months (August 2023 to October 2024), of the 110 isolates of *Acinetobacter spp*, 55 were clinical isolates of MDR *Acinetobacter baumannii* and 55 were patient environmental isolates in whom MDR *Acinetobacter* spp. were isolated. All the clinical isolates included in the study were MDR *Acinetobacter baumannii.*

### 3.1 Characteristics of the study isolates

Among the 55 clinical isolates, 37 (67.3%) were recovered from male patients and 18 (32.7%) from female patients, demonstrating a predominance of MDR *A. baumannii* infections among males. Most clinical isolates were obtained from inpatients (51/55, 92.7%), whereas only four isolates (7.3%) originated from outpatients.

Among the inpatient isolates, 42/51 (82.4%) were recovered from patients admitted to intensive care units (ICUs), while only 9/51 (17.6%) were isolated from patients admitted to general wards. Within the ICUs, the highest number of isolates was obtained from the Critical Care Medical ICU (12/42, 28.6%), followed by the Neuro ICU (8/42, 19.0%), Surgical ICU (7/42, 16.7%), and Medical ICU (6/42, 14.3%). Fewer isolates were recovered from the remaining ICUs, including the Neonatal ICU and Paediatric ICU (2/42, 4.8% each). Among ward admissions, most isolates originated from the private ward (6/9, 66.7%), followed by the male surgical ward (2/9, 22.2%) and the general medicine ward (1/9, 11.1%).

The majority of clinical isolates were recovered from patients aged 61–80 years (19/55, 34.5%), followed by those aged 21–40 years (17/55, 30.9%) and 41–60 years (16/55, 29.1%). Only three isolates (5.5%) were obtained from patients aged ≤20 years.

Endotracheal aspirates represented the most common clinical specimen, accounting for 37/55 (67.3%) isolates, followed by exudates (9/55, 16.4%), sputum (4/55, 7.3%), urine (3/55, 5.5%), and blood (2/55, 3.6%).

Environmental surveillance included 275 samples collected from the surroundings of 55 patients with MDR *A. baumannii* infection. Overall, 67/275 (24.4%) samples yielded MDR *Acinetobacter* spp. Following exclusion of duplicate isolates recovered from multiple sites surrounding the same patient based on identical antimicrobial susceptibility profiles, 55 non-duplicate environmental isolates were included for further phenotypic and genomic analyses. Among the environmental isolates, bed rails accounted for the highest recovery rate (23/55, 41.8%), followed by floor surfaces (20/55, 36.4%), bedside trolleys (12/55, 21.8%), walls (8/55, 14.5%), and nursing station surfaces (4/55, 7.3%).

#### 3.1.1 Antimicrobial susceptibility pattern

All 55 clinical and 55 environmental isolates included in the study were confirmed as multidrug-resistant (MDR) *Acinetobacter baumannii*. Clinical isolates exhibited high levels of resistance to β-lactams, β-lactam/β-lactamase inhibitor combinations, carbapenems, aminoglycosides, fluoroquinolones, and cephalosporins. Among the clinical isolates, tigecycline demonstrated the highest *in vitro* activity, with susceptibility observed in 43/55 (78.2%) isolates, followed by minocycline, which was active against 10/55 (18.2%) isolates. All clinical isolates were reported as intermediate to colistin by the VITEK^®^ 2 system (**Table 1**).

**Table 1:** Antimicrobial susceptibility pattern of clinical MDR *A. baumannii* by Vitek-2 system.

Drug	Sensitive No.	%	Intermediate No.	%	Resistant No.	%	Total No.
Tigecycline	43	78.18	11	20.00	1	1.82	55
Minocycline	10	18.18	6	10.91	39	70.91	55
Gentamycin	6	10.91	2	3.64	47	85.45	55
Cefeperazone/Sulbactam	4	7.27	0	0.00	51	92.73	55
Amikacin	0	0.00	1	1.82	54	98.18	55
Cefepime	0	0.00	3	5.45	52	94.55	55
Levofloxacin	1	1.82	1	1.82	53	96.36	55
Cotrimoxazole	2	3.64	0	0.00	53	96.36	55
Piperacillin/ Tazobactam	0	0.00	0	0.00	55	100.00	55
Ceftazidime	0	0.00	0	0.00	55	100.00	55
Ceftriaxone	0	0.00	0	0.00	55	100.00	55
Imipenem	0	0.00	0	0.00	55	100.00	55
Meropenem	0	0.00	0	0.00	55	100.00	55
Ciprofloxacin	0	0.00	0	0.00	55	100.00	55

Similarly, environmental isolates demonstrated extensive resistance to the routinely tested antimicrobial agents. Tigecycline remained the most active antimicrobial, with susceptibility observed in 30/67 (44.8%) isolates, followed by minocycline, which showed activity against 17/67 (25.4%) isolates (**Table 2**). Based on identical antimicrobial susceptibility profiles, 12 duplicate environmental isolates recovered from multiple sampling sites surrounding the same patient were excluded, resulting in 55 non-duplicate environmental isolates for subsequent phenotypic and genomic analyses.

**Table 2:** Antimicrobial susceptibility pattern of 67 environmental isolates.

Drug	Sensitive No.	%	Intermediate No.	%	Resistant No.	%	Total No.
Tigecycline	30	44.77	26	38.80	11	16.41	67
Minocycline	17	25.3	0	0.00	50	74.62	67
Gentamycin	8	11.91	6	8.95	53	79.1	67
Cefeperazone/Sulbactam	10	14.92	6	8.95	51	76.18	67
Amikacin	0	0.00	10	14.92	57	85.08	67
Cefepime	0	0.00	11	16.41	56	83.64	67
Levofloxacin	0	0.00	10	14.92	57	85.08	67
Cotrimoxazole	3	4.47	0	0.00	64	95.5	67
Piperacillin/Tazobactam	0	0.00	0	0.00	67	100	67
Ceftazidime	0	0.00	0	0.00	67	100	67
Ceftriaxone	0	0.00	0	0.00	67	100	67
Imipenem	0	0.00	0	0.00	67	100	67
Meropenem	0	0.00	0	0.00	67	100	67
Ciprofloxacin	0	0.00	0	0.00	67	100	67

Of the 67 MDR Acinetobacter isolates initially recovered from environmental samples, 55 non-duplicate isolates were included in subsequent comparative phenotypic and genomic analyses.

### 3.2 Phenotypic virulence characteristics

#### 3.2.1 Biofilm formation

Biofilm formation was evaluated using the tissue culture plate assay. Among the clinical isolates, 34/55 (61.8%) were strong biofilm producers, 2/55 (3.6%) were moderate biofilm producers, and 19/55 (34.5%) were classified as non-biofilm producers. In comparison, environmental isolates demonstrated lower biofilm-forming ability, with only 6/55 (10.9%) strong biofilm producers, 19/55 (34.5%) moderate biofilm producers, and 30/55 (54.5%) non-biofilm producers (**Table 3**; **Figure 1**).

**Table 3:** Comparison of Biofilm associated genes among strong, moderate and non-biofilm producing clinical and environmental isolates.

	Clinical isolates (n-55)			Environmental isolates (n=55)		
Biofilm-associated genes	Strong biofilm producer (n=34)	Moderate biofilm producer (n=2)	Non- biofilm producer (n=19)	Strong biofilm producer (n=6)	Moderate biofilm producer (n=19)	Non- biofilm producer (n=30)
*abaI*	34 (100.0%)	2 (100%)	1 (5.3%)	6 (100.0%)	19 (100.0%)	2 (6.7%)
*abaR*	33 (97.1%)	2 (100%)	1 (5.3%)	6 (100.0%)	19 (100.0%)	2 (6.7%)
*Ata*	33 (97.1%)	2 (100%)	0 (0.0%)	6 (100.0%)	18 (94.7%)	2 (6.7%)
*blaPER*	27 (79.4%)	2 (100%)	0 (0.0%)	5 (83.3%)	17 (89.5%)	3 (10.0%)
*Bap*	30 (88.2%)	2 (100%)	1 (5.3%)	5 (83.3%)	16 (84.2%)	2 (6.7%)
*csuA_B*	31 (91.2%)	2 (100%)	0 (0.0%)	6 (100.0%)	18 (94.7%)	2 (6.7%)
*csuB*	31 (91.2%)	2 (100%)	0 (0.0%)	6 (100.0%)	17 (89.5%)	2 (6.7%)
*csuC*	28 (82.4%)	2 (100%)	0 (0.0%)	6 (100.0%)	17 (89.5%)	3 (10.0%)
*csuD*	22 (64.7%)	2 (100%)	0 (0.0%)	4 (66.7%)	16 (84.2%)	3 (10.0%)
*csuE*	28 (82.4%)	2 (100%)	0 (0.0%)	4 (66.7%)	17 (89.5%)	3 (10.0%)
*ompA_3*	34 (100.0%)	2 (100%)	0 (0.0%)	6 (100.0%)	19 (100.0%)	1 (3.3%)
*pgaB*	34 (100.0%)	2 (100%)	2 (10.5%)	6 (100.0%)	19 (100.0%)	5 (16.7%)
*pgaC*	34 (100.0%)	2 (100%)	2 (10.5%)	6 (100.0%)	19 (100.0%)	4 (13.3%)
*pgaD*	34 (100.0%)	2 (100%)	2 (10.5%)	6 (100.0%)	19 (100.0%)	5 (16.7%)

**Figure 1:**
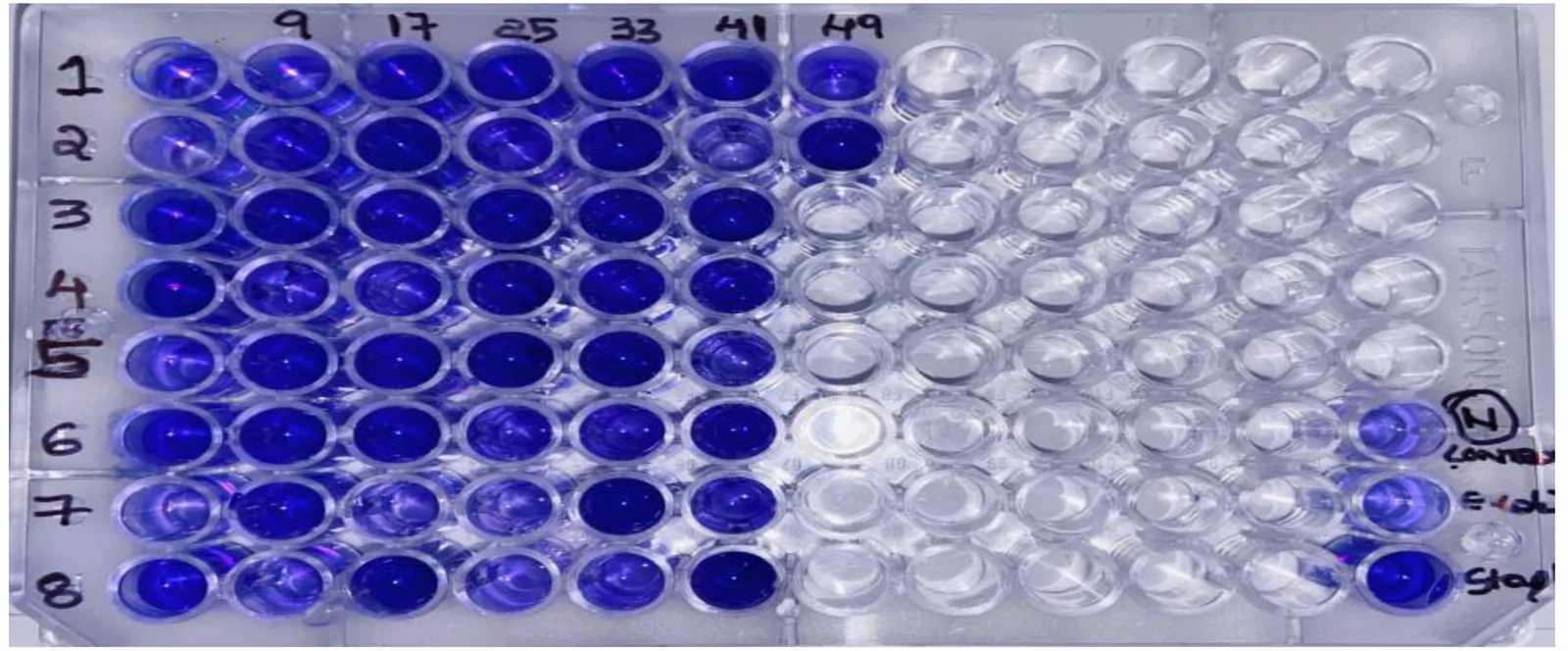
Biofilm production by Tissue Culture Plate Method with ATCC *S. aureus* 25923 (positive) & ATCC *E. coli* 25922 as (negative) control.

#### 3.2.2 Haemolytic activity

Haemolytic activity was detected in 29/55 (52.7%) clinical isolates compared with 13/55 (23.6%) environmental isolates, whereas 26/55 (47.3%) clinical isolates and 42/55 (76.4%) environmental isolates were non-haemolytic (**Table 4****;** **Figure 2**).

**Table 4:** Distribution of Haemolysis-associated genes.

Haemolysis-associated genes	Clinical isolates (n=55)		Environmental isolates (n=55)	
	Haemolytic (n=29)	Non-haemolytic (n=26)	Haemolytic (n=13)	Non-Haemolytic (n=42)
*hemO (heme/iron acquisition)*	29 (100%)	0	11 (84.6%)	2 (4.76%)
*plc1 (Phospholipase C)*	19 (65.5%)	0	11 (76.9%)	3 (7.14%)
*plc2 (Phospholipase C)*	26 (89.65%)	4 (15.38%)	9 (69.23%)	1 (2.38%)
*plcD (Phospholipase D)*	16 (55.17%)	2 (7.69%)	11 (84.6%)	0

**Figure 2:**
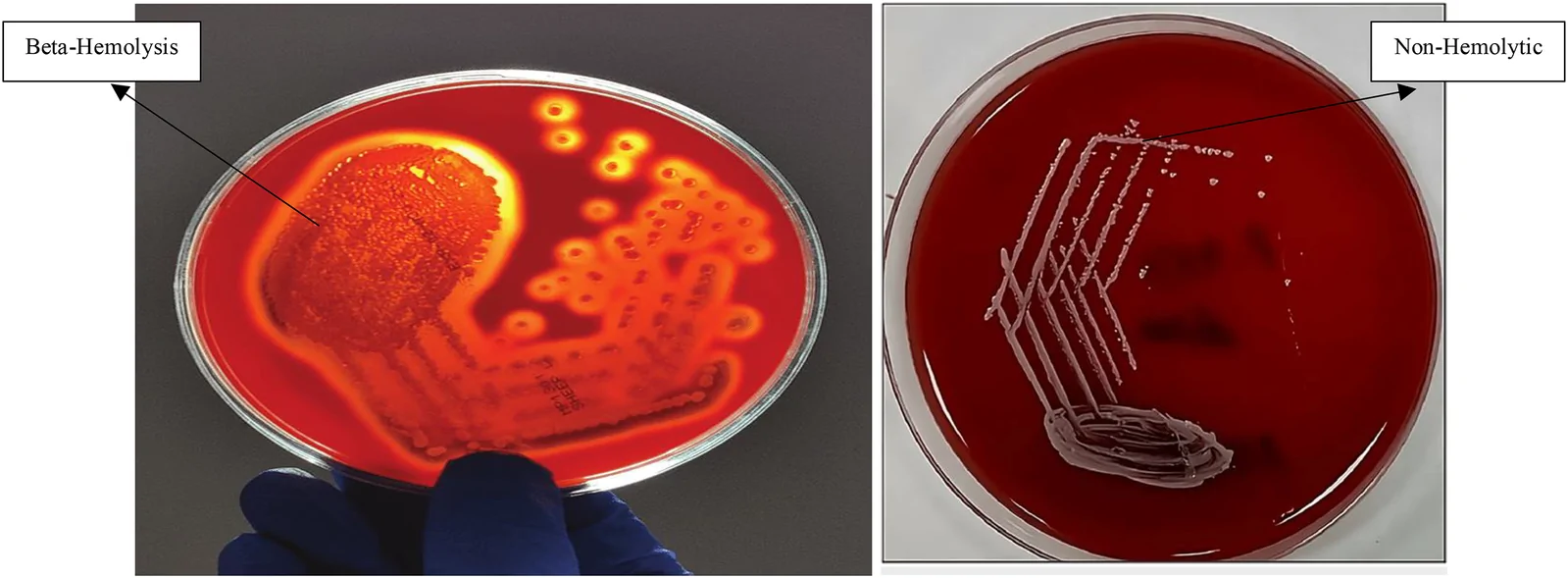
Hemolytic activity on Sheep Blood Agar .

#### 3.2.3 Proteolytic activity

Proteolytic activity was observed in 36/55 (65.5%) clinical isolates and 33/55 (60.0%) environmental isolates, while 19/55 (34.5%) clinical isolates and 22/55 (40.0%) environmental isolates lacked proteolytic activity (**Table 5**; **Figure 3**).

**Table 5:** Distribution of Proteolysis-associated genes.

Proteolysis-associated genes	Clinical isolates (n=55)		Env. isolates (n=55)	
	Proteolytic (n=36)	Non- proteolytic (n=19)	Proteolytic (n=33)	Non- proteolytic (n=22)
*ata* *(Autotransporter adhesin)*	33 (91.66%)	0	33 (100%)	0
*cpaA* *(Zinc-metalloprotease)*	34 (94.4%)	0	33 (100%)	1 (4.5%)

**Figure 3:**
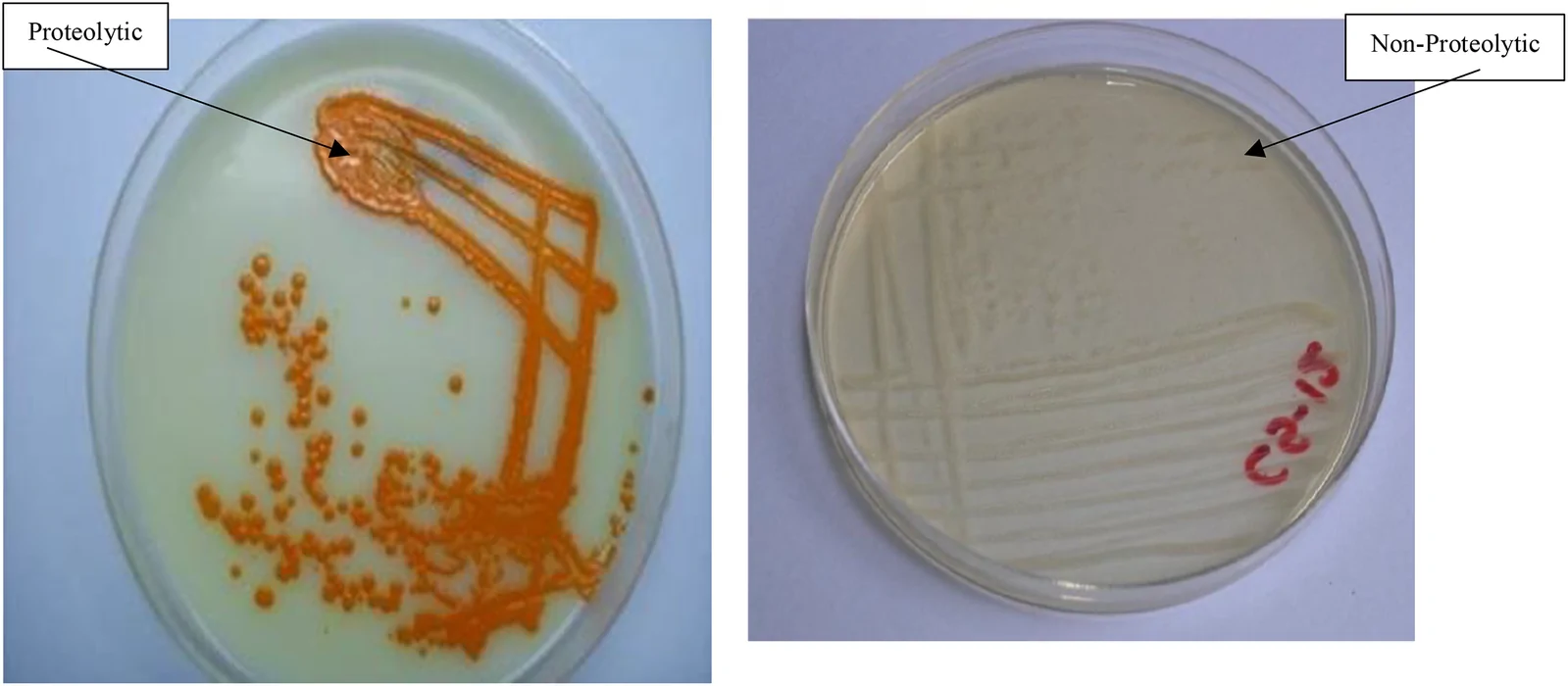
Results of proteolytic activity on skim milk agar.

#### 3.2.4 Siderophore production

Siderophore production was demonstrated by 31/55 (56.4%) clinical isolates and 17/55 (30.9%) environmental isolates. The remaining 24/55 (43.6%) clinical isolates and 38/55 (69.1%) environmental isolates did not produce siderophores on Chrome Azurol S agar (**Table 6**; **Figure 4**).

**Table 6:** Distribution of siderophore associated-genes.

Siderophore associated Genes	Clinical isolates (n=55)		Environmental isolates (n=55)	
	Siderophore positive (n=31)	Siderophore negative (n=24)	Siderophore positive (n=17)	Siderophore negative (n=38)
barA	31 (100%)	1 (4.2%)	17 (100%)	2 (5.3%)
barB	31 (100%)	1 (4.2%)	17 (100%)	2 (5.3%)
basA	30 (96.8%)	1 (4.2%)	17 (100%)	2 (5.3%)
basB	31 (100%)	0 (0%)	16 (94.1%)	0 (0%)
basC	31 (100%)	0 (0%)	17 (100%)	0 (0%)
basD	30 (96.8%)	0 (0%)	16 (94.1%)	0 (0%)
basF	31 (100%)	0 (0%)	17 (100%)	0 (0%)
basG	31 (100%)	1 (4.2%)	17 (100%)	1 (2.6%)
basH	30 (96.8%)	0 (0%)	16 (94.1%)	0 (0%)
basI	28 (90.3%)	0 (0%)	15 (88.2%)	0 (0%)
basJ	31 (100%)	0 (0%)	17 (100%)	0 (0%)
bauA	31 (100%)	0 (0%)	16 (94.1%)	0 (0%)
bauB	31 (100%)	0 (0%)	17 (100%)	0 (0%)
bauC	30 (96.8%)	0 (0%)	16 (94.1%)	0 (0%)
bauD	31 (100%)	0 (0%)	17 (100%)	0 (0%)
bauE	30 (96.8%)	4 (16.7%)	17 (100%)	5 (13.2%)
bauF	31 (100%)	6 (25.0%)	17 (100%)	6 (15.8%)
entE	31 (100%)	2 (8.3%)	17 (100%)	3 (7.9%)

**Figure 4:**
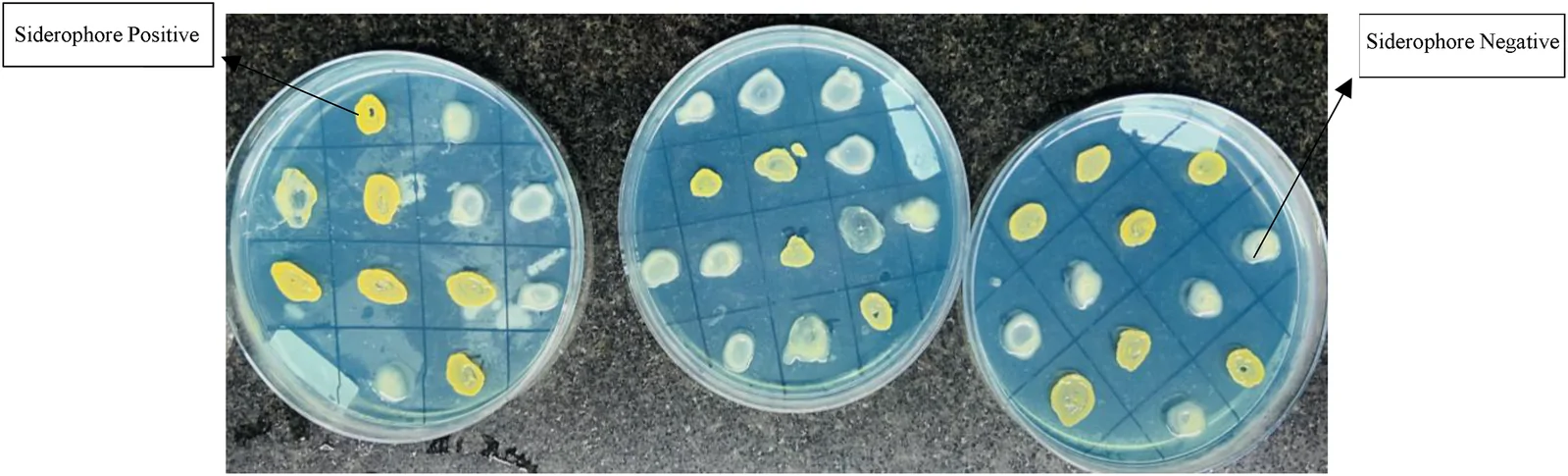
Results of siderophore production on chrome azurol S agar.

### 3.3 Distribution of virulence-associated genes

#### 3.3.1 Biofilm-associated genes

Among the 34 strong biofilm-producing clinical isolates, *abaI*, *ompA*, *pgaB*, *pgaC*, and *pgaD* were detected in all isolates (34/34, 100%). *abaR* and *ata* were identified in 33/34 (97.1%) isolates, *bap* in 30/34 (88.2%), *csuA/B* and *csuB* in 31/34 (91.2%), *csuC* and *csuE* in 28/34 (82.4%), and *csuD* in 22/34 (64.7%). Among the two moderate biofilm producers, *abaI*, *ompA*, *pgaB*, *pgaC*, and *pgaD* were detected in both isolates (2/2, 100%). In contrast, non-biofilm-producing isolates showed markedly lower carriage of biofilm-associated genes (**Table 3**).

Among environmental isolates, all six strong biofilm producers carried *abaI*, *abaR*, *ata*, *csuA/B*, *csuB*, *csuC*, *ompA*, *pgaB*, *pgaC*, and *pgaD* (6/6, 100%), whereas *bap* and *blaPER* were detected in 5/6 (83.3%) isolates. Moderate biofilm-producing isolates also demonstrated a high prevalence of biofilm-associated genes, whereas non-biofilm producers exhibited substantially lower frequencies (**Table 3**).

#### 3.3.2 Haemolysis-associated genes

Among haemolytic clinical isolates, *hemO* was detected in all isolates (29/29, 100%), followed by *plc2* in 26/29 (89.7%), *plc1* in 19/29 (65.5%), and *plcD* in 16/29 (55.2%). In comparison, these genes were identified only sporadically among non-haemolytic clinical isolates. Environmental haemolytic isolates similarly demonstrated a high prevalence of *hemO*(11/13, 84.6%), *plc1* (10/13, 76.9%), *plc2* (9/13, 69.2%), and *plcD* (11/13, 84.6%), whereas gene carriage among non-haemolytic environmental isolates remained low (**Table 4**).

#### 3.3.3 Proteolysis-associated genes

Among proteolytic clinical isolates, *cpaA* was detected in 34/36 (94.4%) isolates and *ata* in 33/36 (91.7%). Neither gene was identified among non-proteolytic clinical isolates. Similarly, all 33 proteolytic environmental isolates carried both *ata*(33/33, 100%) and *cpaA* (33/33, 100%), whereas *cpaA* was detected in only 1/22 (4.5%) non-proteolytic environmental isolate (**Table 5**).

#### 3.3.4 Siderophore-associated genes

Among siderophore-producing clinical isolates, *barA*, *barB*, *basB*, *basC*, *basF*, *basG*, *basJ*, *bauA*, *bauB*, *bauD*, *bauF* and *entE* were detected in all isolates (31/31, 100%), whereas *basA*, *basD*, *basH*, *bauC*, and *bauE* were identified in 30/31 (96.8%) isolates and *basI* in 28/31 (90.3%). Environmental siderophore-producing isolates similarly demonstrated a high prevalence of genes belonging to the *bar*, *bas*, *bau*, and *ent* clusters, although the overall frequency was lower than that observed among clinical isolates (**Table 6**) (**Figures 5**, **6**).

**Figure 5:**
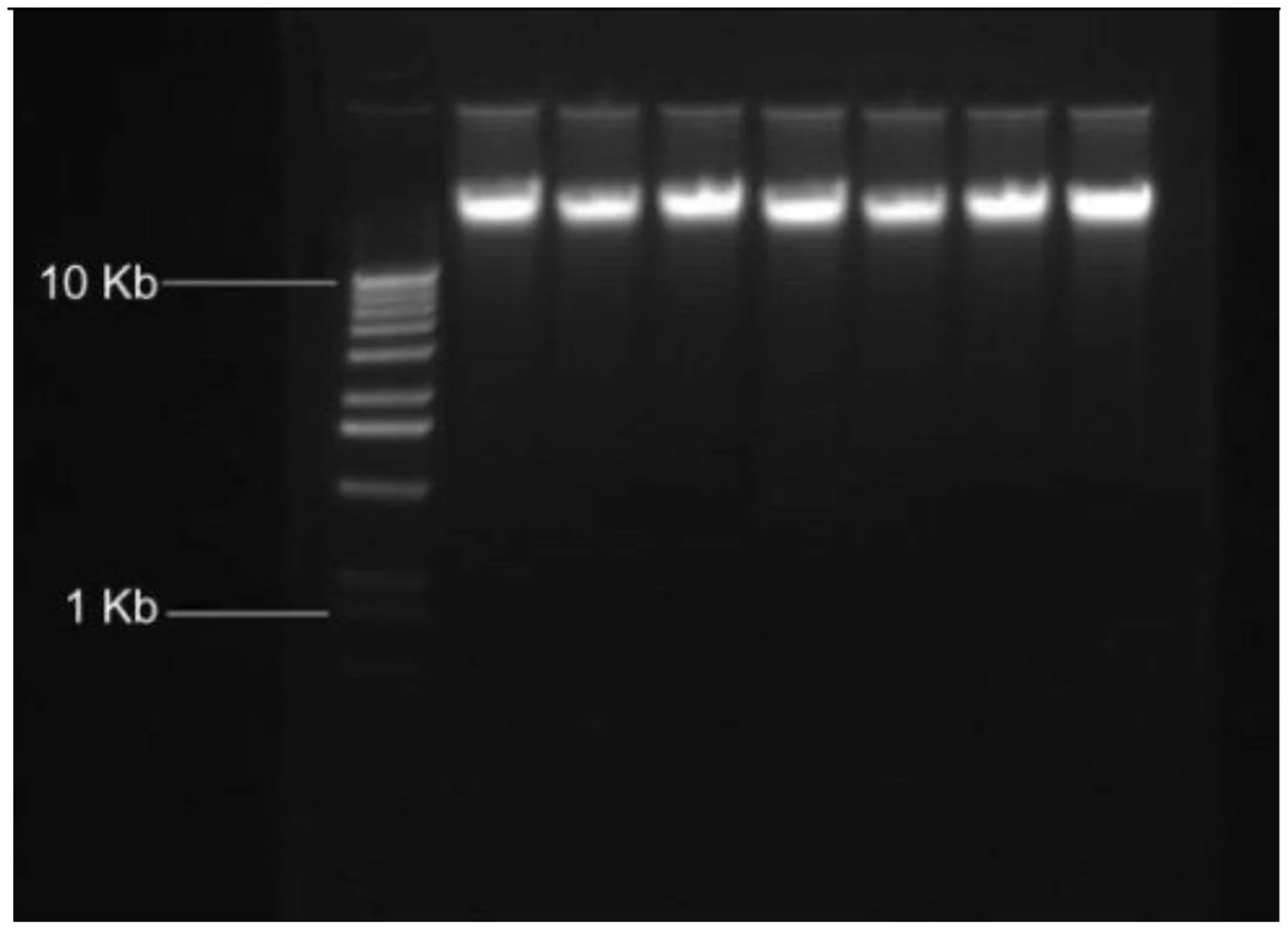
Agarose gel electrophoresis showing genomic DNA extracted by the WASH method for Whole Genome Sequencing (WGS). Lane M: 1 kb DNA ladder; Lanes 1–7: genomic DNA of bacterial isolates showing high-molecular-weight DNA bands near the 10 kb region indicating.

**Figure 6:**
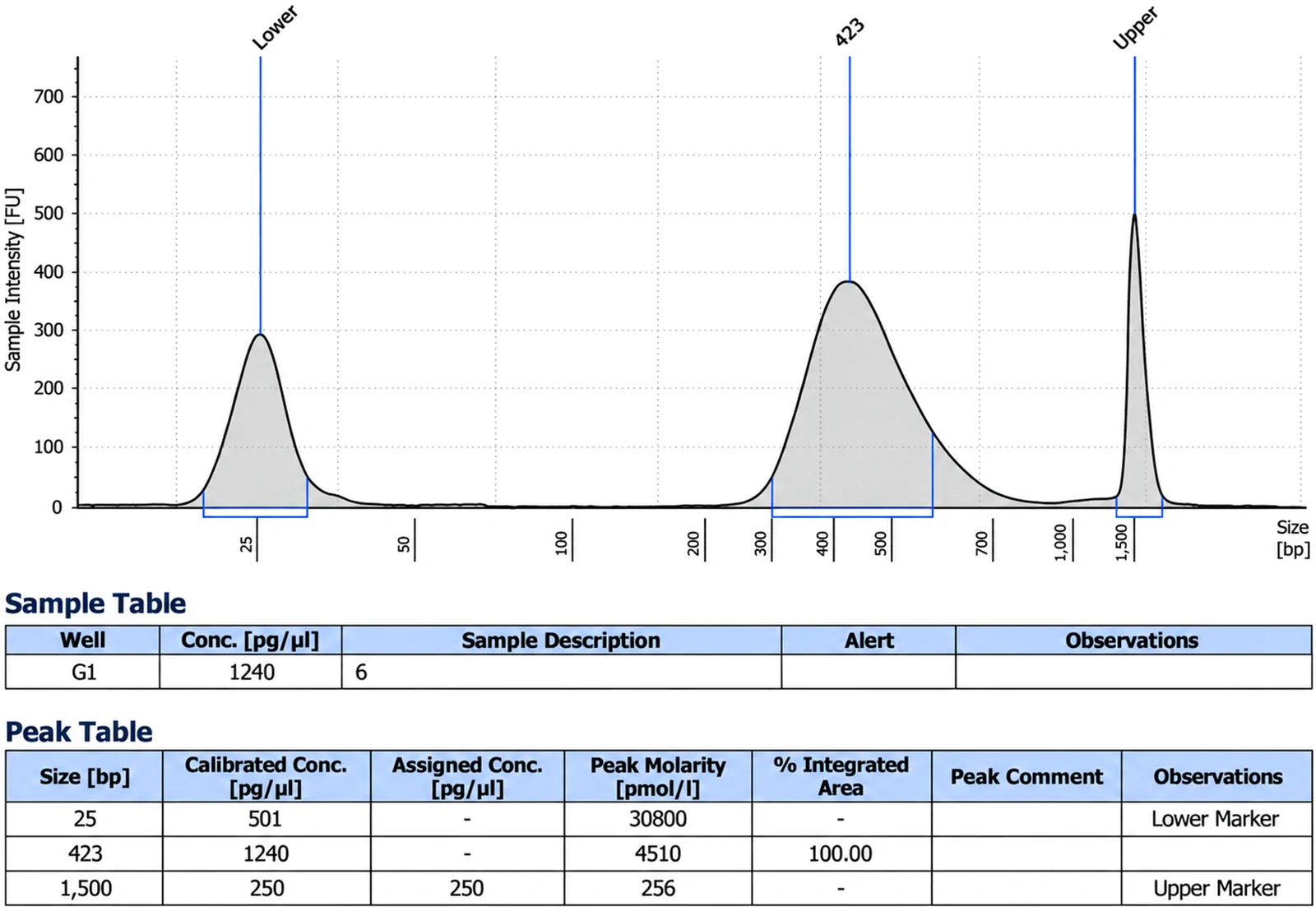
Fragment analyzer electropherogram of DNA library prepared for Whole Genome Sequencing showing a major peak at ~423 bp with lower (25 bp) and upper (1500 bp) markers.

### 3.4 Comparative analysis of clinical and environmental isolates

Clinical isolates demonstrated significantly greater virulence than environmental isolates. Strong biofilm formation was significantly more frequent among clinical isolates (34/55, 61.8%) than environmental isolates (6/55, 10.9%) (*P* < 0.0001). Likewise, haemolytic activity (29/55, 52.7% vs. 13/55, 23.6%; *P* = 0.0032) and siderophore production (31/55, 56.4% vs. 17/55, 30.9%; *P* = 0.0124) were significantly higher among clinical isolates. In contrast, proteolytic activity did not differ significantly between clinical (36/55, 65.5%) and environmental isolates (33/55, 60.0%; *P* = 0.6933) (**Table 7**).

**Table 7:** Statistical analysis of phenotypic virulence attributes.

Virulence attribute	Phenotype	Clinical isolates (n = 55)	Env. isolates (n = 55)	χ² test p-value	Statistical significance
Biofilm formation	Strong biofilm producers	34 (61.81%)	6 (10.90%)	< 0.0001	Highly significant
	Moderate biofilm producers	2 (5.45%)	19 (34.54%)		
	Non-biofilm producers	19 (32.72%)	30 (54.54%)		
Haemolytic activity	Beta-haemolytic	29 (52.7%)	13 (23.6%)	0.0032	Significant
	Non-haemolytic	26 (47.3%)	42 (76.4%)		
Proteolytic activity	Proteolytic	36 (65.5%)	33 (60.0%)	0.6933	Not significant
	Non-proteolytic	19 (34.5%)	22 (40.0%)		
Siderophore production	Siderophore-positive	31 (56.4%)	17 (30.9%)	0.0124	Significant
	Siderophore-negative	24 (43.6%)	38 (69.1%)		

Comparison of virulence-associated genes demonstrated significantly higher frequencies of *abaI*, *abaR*, *ata*, *bap*, *ompA*, *pgaB*, *pgaC*, *pgaD*, *hemO*, *plc2*, *barA*, *barB*, *basA*, and *entE* among clinical isolates than environmental isolates (*P* < 0.05). No statistically significant differences were observed for several other biofilm-, haemolysis-, proteolysis-, and siderophore-associated genes (**Table 8**).

**Table 8:** Distribution & statistical analysis of virulence-associated genes.

Virulence category	Gene	Clinical isolates	Env. isolates	P-value	Significance
Biofilm-associated genes	*abaI*	37 (67.3%)	27 (49.1%)	0.041	Significant
	*abaR*	36 (65.5%)	27 (49.1%)	0.048	Significant
	*Ata*	35 (63.6%)	26 (47.3%)	0.047	Significant
	*blaPER*	29 (52.7%)	25 (45.5%)	0.430	Not significant
	*Bap*	33 (60.0%)	23 (41.8%)	0.036	Significant
	*csuA_B*	33 (60.0%)	26 (47.3%)	0.170	Not significant
	*csuB*	33 (60.0%)	25 (45.5%)	0.120	Not significant
	*csuC*	30 (54.5%)	26 (47.3%)	0.450	Not significant
	*csuD*	24 (43.6%)	23 (41.8%)	0.850	Not significant
	*csuE*	30 (54.5%)	24 (43.6%)	0.240	Not significant
	*ompA_3*	36 (65.5%)	26 (47.3%)	0.032	Significant
	*pgaB*	38 (69.1%)	30 (54.5%)	0.048	Significant
	*pgaC*	38 (69.1%)	29 (52.7%)	0.041	Significant
	*pgaD*	38 (69.1%)	30 (54.5%)	0.048	Significant
Haemolysis-associated genes	*hemO*	29 (52.7%)	13 (23.6%)	0.002	Significant
	*plc1*	19 (34.5%)	11 (20.0%)	0.087	Not significant
	*plc2*	30 (54.5%)	10 (18.2%)	<0.0001	Significant
	*plcD*	18 (32.7%)	11 (20.0%)	0.120	Not significant
Proteolysis-associated genes	*ata*	33 (60.0%)	33 (60.0%)	1.000	Not significant
	*cpaA*	34 (61.8%)	34 (61.8%)	1.000	Not significant
Siderophore-associated genes	*barA*	32 (58.2%)	19 (34.5%)	0.011	Significant
	*barB*	32 (58.2%)	19 (34.5%)	0.011	Significant
	*basA*	31 (56.4%)	19 (34.5%)	0.021	Significant
	*basB*	31 (56.4%)	16 (29.1%)	0.065	Not significant
	*basC*	31 (56.4%)	17 (30.9%)	0.058	Not significant
	*basD*	30 (54.5%)	16 (29.1%)	0.060	Not significant
	*basF*	31 (56.4%)	17 (30.9%)	0.058	Not significant
	*basG*	32 (58.2%)	18 (32.7%)	0.064	Not significant
	*basH*	30 (54.5%)	16 (29.1%)	0.060	Not significant
	*basI*	28 (50.9%)	15 (27.3%)	0.072	Not significant
	*basJ*	31 (56.4%)	17 (30.9%)	0.058	Not significant
	*bauA*	31 (56.4%)	16 (29.1%)	0.065	Not significant
	*bauB*	31 (56.4%)	17 (30.9%)	0.058	Not significant
	*bauC*	30 (54.5%)	16 (29.1%)	0.060	Not significant
	*bauD*	31 (56.4%)	17 (30.9%)	0.058	Not significant
	*bauE*	34 (61.8%)	22 (40.0%)	0.074	Not significant
	*bauF*	37 (67.3%)	23 (41.8%)	0.062	Not significant
	*entE*	33 (60.0%)	20 (36.4%)	0.014	Significant

Overall, clinical MDR *A. baumannii* isolates exhibited significantly greater phenotypic virulence and a higher prevalence of several virulence-associated genes than environmental isolates, although environmental isolates also retained multiple virulence determinants, highlighting their potential role as reservoirs within the hospital environment.

Yes. Using your exact 12 references, I would cite the Discussion like this. I have not added unnecessary references to your own results; citations are attached only to statements supported by published literature.

## 4 Discussion

Multidrug-resistant (MDR) *Acinetobacter baumannii* is an important healthcare-associated pathogen because of its ability to combine extensive antimicrobial resistance with environmental persistence and multiple virulence-associated traits (Wong et al., 2017; Harding et al., 2018). The present study compared the phenotypic and genomic virulence profiles of clinical and patient-environmental MDR *A. baumannii* isolates from a tertiary care hospital in South India. The major finding was that clinical isolates exhibited significantly higher frequencies of strong biofilm formation, haemolytic activity, and siderophore production, while environmental isolates retained several virulence-associated determinants despite their lower phenotypic expression.

The predominance of clinical isolates from male patients and critically ill individuals, particularly those admitted to ICUs, is consistent with the established epidemiology of MDR *A. baumannii*. The high proportion of isolates recovered from endotracheal aspirates further reflects the association of this pathogen with severe respiratory infections and critically ill patients. Prolonged hospitalization, invasive procedures, antimicrobial exposure, and other ICU-related factors are recognized as important risk factors for MDR and extensively drug-resistant *A. baumannii* infection (Diao et al., 2024). The ability of *A. baumannii* to withstand environmental stresses and persist outside the host further contributes to its successful establishment in healthcare settings (Harding et al., 2018; Monem et al., 2020).

Environmental surveillance identified MDR *Acinetobacter* in 24.4% of the samples collected from patient surroundings, with bed rails and floor surfaces accounting for the largest proportion of recoveries. Although environmental recovery does not establish directionality of transmission, these findings support the role of contaminated hospital surfaces as potential reservoirs for MDR *A. baumannii* (Weber et al., 2010; Monem et al., 2020). Importantly, the environmental isolates in this study retained several virulence-associated genes, suggesting that environmental persistence and pathogenic potential are not mutually exclusive.

### 4.1 Biofilm formation

The most pronounced difference between clinical and environmental isolates was observed for biofilm formation. Strong biofilm production was detected in 61.8% of clinical isolates compared with only 10.9% of environmental isolates. Biofilm formation is an important survival mechanism of *A. baumannii* and contributes to persistence on abiotic surfaces and medical devices (Espinal et al., 2012; Harding et al., 2018).

The high prevalence of *abaI*, *ompA*, *pgaB*, *pgaC*, and *pgaD* among strong biofilm-producing clinical isolates further supports the multifactorial nature of biofilm formation. The *pgaABCD* locus contributes to production of poly-β-1,6-N-acetylglucosamine, an important component of the biofilm matrix (Choi et al., 2009). OmpA and the Csu pilus system are also involved in adhesion and biofilm development (Harding et al., 2018; Mea et al., 2021). The high prevalence of *abaI* and *abaR* in the present study is consistent with the involvement of the AbaI/AbaR quorum-sensing system in regulation of biofilm-associated phenotypes (Oh and Han, 2020).

However, the presence of biofilm-associated genes in some non-biofilm-producing isolates highlights an important genotype–phenotype distinction. Gene carriage represents genetic potential, whereas phenotypic expression depends on regulatory mechanisms, environmental conditions, and interactions among multiple determinants. Thus, biofilm formation cannot be reliably predicted from the presence of individual genes alone.

### 4.2 Haemolytic activity

Haemolytic activity was significantly higher among clinical isolates than environmental isolates. The higher prevalence of *hemO*, *plc1*, *plc2*, and *plcD* among haemolytic isolates provides biological support for their potential contribution to host-associated pathogenicity. Virulence in *A. baumannii* involves multiple interacting mechanisms, and haemolytic and membrane-associated activities may contribute to host tissue damage and nutrient acquisition (Wong et al., 2017; Harding et al., 2018). However, the incomplete correspondence between gene carriage and haemolytic phenotype indicates that gene expression, regulation, and other interacting factors may influence the observed phenotype.

### 4.3 Proteolytic activity and genotype–phenotype relationship

Unlike the other phenotypes, proteolytic activity did not differ significantly between clinical and environmental isolates. The high prevalence of *cpaA* and *ata* among proteolytic isolates suggests that proteolytic capacity may represent a relatively conserved trait within the studied population. CpaA is a secreted protease associated with *A. baumannii* clinical isolates and has been shown to interfere with host coagulation through degradation of coagulation-associated proteins (Tilley et al., 2014).

The incomplete concordance observed between virulence genes and phenotypic expression is biologically important. Whole-genome sequencing identifies the repertoire of potential virulence determinants, whereas phenotypic assays provide evidence of functional expression under specific laboratory conditions. Combining both approaches therefore provides a more comprehensive assessment of *A. baumannii* virulence.

### 4.4 Siderophore production

Siderophore production was significantly higher among clinical isolates than environmental isolates (56.4% vs. 30.9%), highlighting the potential importance of iron acquisition in host-associated adaptation. Iron is essential for bacterial growth but is strongly restricted within the host as part of nutritional immunity. *A. baumannii* possesses multiple siderophore-mediated iron-acquisition systems, including acinetobactin and baumannoferrin, which facilitate iron acquisition under iron-limited conditions (Dorsey et al., 2004; Wong et al., 2017; Sheldon and Skaar, 2020).

The high prevalence of *bar*, *bas*, *bau*, and *entE* genes among siderophore-producing clinical isolates supports the importance of these systems in maintaining iron acquisition during infection. The *bas* and *bau* gene clusters are involved in acinetobactin biosynthesis and uptake, while associated transport systems facilitate utilization of iron-bound siderophores (Dorsey et al., 2004; Sheldon and Skaar, 2020). The significantly higher frequency of selected siderophore-associated genes among clinical isolates may therefore contribute to their enhanced adaptation to the host environment.

Nevertheless, environmental isolates also retained several siderophore-associated determinants, indicating that environmental strains possess substantial genetic potential for iron acquisition. The lower phenotypic expression observed among environmental isolates may reflect differences in ecological conditions or regulation rather than complete loss of this virulence-associated capacity. Thus, siderophore-mediated iron acquisition may contribute both to host adaptation and to the persistence of MDR *A. baumannii* within the hospital environment (Monem et al., 2020; Sheldon and Skaar, 2020).

### 4.5 Clinical and environmental significance

The higher frequency of selected virulence phenotypes and genes among clinical isolates suggests that host-associated conditions may favour strains or phenotypes with enhanced biofilm formation, haemolysis, and iron acquisition. Nevertheless, environmental isolates retained multiple virulence determinants, demonstrating that hospital environmental reservoirs may harbour organisms with considerable pathogenic potential.

These findings have implications for infection prevention and control. The recovery of MDR *A. baumannii* from frequently touched surfaces, together with the presence of virulence-associated determinants, emphasizes the importance of environmental cleaning, hand hygiene, appropriate disinfection, and surveillance of high-risk areas (Weber et al., 2010; Monem et al., 2020). However, environmental contamination alone cannot establish transmission; longitudinal sampling and high-resolution genomic epidemiology are required to determine whether environmental strains directly contribute to subsequent clinical infections.

### 4.6 Strengths and limitations

The major strength of this study is the integrated assessment of clinical and patient-environmental isolates using both phenotypic virulence assays and whole-genome sequencing. Nevertheless, the single-centre design and relatively modest sample size may limit generalizability. In addition, detection of virulence genes does not establish their expression or functional activity, and the cross-sectional environmental sampling design cannot determine the direction of transmission between patients and their surroundings.

Overall, the findings demonstrate that clinical and environmental MDR *A. baumannii* isolates possess overlapping but quantitatively different virulence profiles. Clinical isolates showed greater expression of selected virulence traits, whereas environmental isolates retained a substantial repertoire of virulence-associated genes. The coexistence of antimicrobial resistance, environmental persistence, and virulence potential highlights the importance of considering both patient-associated and environmental reservoirs in the control of MDR *A. baumannii* in healthcare settings (Wong et al., 2017; Harding et al., 2018; Monem et al., 2020).

## 5 Conclusion

This study demonstrated that multidrug-resistant (MDR) *Acinetobacter baumannii* clinical isolates possess greater phenotypic virulence and a higher prevalence of virulence-associated genes than corresponding patient-environmental isolates. However, environmental isolates retained multiple virulence determinants, highlighting their potential role as reservoirs for persistence and transmission within healthcare settings. The observed phenotype–genotype discordance indicates that the presence of virulence-associated genes alone does not necessarily predict phenotypic expression. Overall, integrating phenotypic virulence assays with whole-genome sequencing provides a comprehensive understanding of the virulence potential of MDR *A. baumannii* and supports improved infection prevention, environmental surveillance, and control strategies in healthcare facilities.

## Data Availability

The names of the repository/repositories and accession number(s) can be found below: https://www.ncbi.nlm.nih.gov/, PRJEB50614, https://www.ncbi.nlm.nih.gov/, PRJEB50614.
